# Spartan Face Mask Detection and Facial Recognition System

**DOI:** 10.3390/healthcare10010087

**Published:** 2022-01-03

**Authors:** Ziwei Song, Kristie Nguyen, Tien Nguyen, Catherine Cho, Jerry Gao

**Affiliations:** 1 Department of Applied Data Science, San Jose State University, San Jose, CA 95192, USA; ziwei.song@sjsu.edu (Z.S.); Kristie.nguyen@sjsu.edu (K.N.); tien.t.nguyen01@sjsu.edu (T.N.); catherine.cho@sjsu.edu (C.C.); 2 Department of Computer Engineering, San Jose State University, San Jose, CA 95192, USA

**Keywords:** COVID-19, masked face, facial recognition, deep learning, ensemble model

## Abstract

According to the World Health Organization (WHO), wearing a face mask is one of the most effective protections from airborne infectious diseases such as COVID-19. Since the spread of COVID-19, infected countries have been enforcing strict mask regulation for indoor businesses and public spaces. While wearing a mask is a requirement, the position and type of the mask should also be considered in order to increase the effectiveness of face masks, especially at specific public locations. However, this makes it difficult for conventional facial recognition technology to identify individuals for security checks. To solve this problem, the Spartan Face Detection and Facial Recognition System with stacking ensemble deep learning algorithms is proposed to cover four major issues: Mask Detection, Mask Type Classification, Mask Position Classification and Identity Recognition. CNN, AlexNet, VGG16, and Facial Recognition Pipeline with FaceNet are the Deep Learning algorithms used to classify the features in each scenario. This system is powered by five components including training platform, server, supporting frameworks, hardware, and user interface. Complete unit tests, use cases, and results analytics are used to evaluate and monitor the performance of the system. The system provides cost-efficient face detection and facial recognition with masks solutions for enterprises and schools that can be easily applied on edge-devices.

## 1. Introduction

According to the CDC COVID Data tracker, over 30,000,000 cases have been confirmed since January 2020 in the US. As one of the most effective protections from COVID-19, wearing a mask is required and strongly recommended by CDC. To effectively reduce the spread of COVID-19, wearing masks that completely cover the mouth and nose is required for public transportation and indoor businesses. Thus, face detection and recognition with masks on are crucial and urgent tasks to many enterprises and institutions. Artificial intelligence and computer vision techniques are widely used for facial recognition and face detection with occlusions.

From the Mordor Intelligence market report, the facial recognition market was valued at 3.72 billion USD in 2020 and is expected at a compound annual growth rate (CAGR) of 21.71% over the forecast period 2021 to 2026 [1]. Given the scale of the pandemic, growing demand for facial recognition systems based on AI platforms among enterprises, hospitals, and schools is the key trend of the market. Most of the systems use AI technology and machine learning algorithms to detect faces, identify and verify a person from a digital image or video source. These systems are mainly used for access control, security and surveillance. Current leading companies in the field of facial recognition services provide cloud-based applications and systems for large enterprises that are along with their existing CCTV or surveillance cameras. However, adoption of cost-efficient face detection and facial recognition services among small and medium-sized enterprises (SMEs) is also a substantial growing segment [2]. Within the segment, face detection and facial recognition solutions are not providing detailed access check such as mask type detection and mask position detection which are two important access conditions in response to the COVID-19. Additional requirements on the systems are adaptability to physical access control systems using internet protocol (IP) cameras or other end-devices cameras.

In this paper, we propose a camera-based Spartan Face Mask detection and Facial Recognition System consisting of four features: (1) mask detection, (2) mask position detection, (3) mask type detection, and (4) facial recognition with mask. Frames of videos captured from built-in cameras or surveillance cameras are the input, and they are processed to satisfy the functional requirements as following:

Mask detection: divide the captured face into regions of interest to detect the feature (mask) present in the regions of interest, and display if a person is wearing a mask or not.

Mask position detection: use regions of interest to detect four types of mask positions including under nose, under mouth, under chin, and correct wear.

Mask type: use regions of interest to detect three types of masks including N95s, surgical masks, and cloth coverings.

Facial recognition with mask on: identify personnel automatically without removing the face mask and provide matching identification results from a designated database.

We introduce a stacking ensemble model learning framework that is based on machine learning feature extraction, deep learning models, and transfer learning as the key algorithm support for the system. Modified CNN, AlexNet, and VGG16 are applied for different tasks of mask detection. With respect to facial recognition tasks, we develop a face mask facial recognition pipeline with improved FaceNet as face detector and modified SVM and Xgboost as classifiers. The framework processes the models in parallel, which boosts the overall prediction accuracy and it is easier to modify each feature.

The novelty of this research is providing an end-to-end comprehensive system that supports face mask detection, mask position detection, mask type detection, and facial recognition with masks. This Spartan system is built with five components which are training platform, server, supporting framework, hardware, and interface. Training platform provides the model training process with model evaluation and unit tests for each model. The server is powered by the stacking ensemble model learning framework along with video processor and prediction generation. Supporting framework combines all open-source softwares or platforms that are applied in the system. Hardware supporting this system is mainly edge devices with cameras. User interface is designed to display real-time video streaming, predictions and access status.

This solution can be easily integrated with surveillance cameras, and generate reliable real-time predictions that automates the mask detection process. In addition, the comprehensive Spartan system is portable and lightweight since the source code takes less than 1 GB. Therefore, it is applicable for small businesses, colleges, and universities for efficient access check during the pandemic and helps to follow social distancing guidelines.

The outline of the rest of the paper is as follows. Section 2 reviews the related work and technologies, Section 3 describes the characteristics of the dataset, Section 4 describes the methodology and justification, Section 5 analyzes the model results with case study, Section 6 presents details of system design, Section 7 presents system evaluation and visualization, and Section 8 discusses conclusions and future work.

## 2. Related Work

### 2.1. Literature Survey

For face recognition and facial feature extraction, many algorithms and deep learning models are proposed by other studies to detect faces with different kinds of occlusions. For image classification problems, traditional machine learning algorithms and deep neural networks models such as CNNs, SVM, and decision trees are widely used. Additionally, many studies have used combined or hybrid approaches for mask detection and facial recognition problems. Table 1 summarizes some studies and compares each study by its focused problem, algorithms, accuracy, data and labeling methods.

According to Loey et al., hybrid models are applied to a two-step facial recognition process [3]. First step is feature extraction with ResNet 50, and the second step is classification with ensemble machine learning algorithms such as Support Vector Machine (SVM) and decision trees. The overall classification accuracy achieves 99.49%. Besides using facial feature extraction to classify human faces, some studies proposed alternative solutions where incomplete face features are used for face detection. For example, one of the solutions is to use Mask Projection techniques in order to simulate incomplete facial features [4]. Another solution is to utilize part of the facial features, especially the feature of the eye, for facial recognition [5].

On the consideration of mask type classification, Wang [6] implemented deep learning techniques to identify four types of masks: bare, homemade, surgical, and N95. Deep learning algorithm Ssd_mobilenet_v2_coco was used to classify the four types of masks with an accuracy of 97%. The model was then deployed to a Raspberry Pi, which created a bounding box around the individual’s face with a display of the correct mask type class. Ge et al. [7] used LLE-CNNs to classify the mask type on static images with an accuracy of 76.4%.

In terms of proper mask wearing detection, He et al. [8] proposed facial attribute recognition using synthesized abstraction images which achieved better attribute prediction results compared with using original images. The process was implemented in two steps. First step was to generate abstraction facial images by Generative Adversarial Networks (GAN). Next, the abstracted images along with the originals were fed into a dual-path facial Attribute Prediction Net. This proposed framework was evaluated on two benchmarks, CelebA and LFWA face datasets. Their results outperformed the state-of-art methods. Song et al. [9] found that traditional deep CNN networks performed poorly over occlusion face recognition problems. Thus, they proposed a mask learning strategy based on a trunk CNN model trained for face recognition to find and discard feature elements from recognition. They established the pairwise differential siamese network (PDSN) structure to learn the correspondence between occluded facial blocks and corrupted feature elements. Then, a mask dictionary was established accordingly which was used to composite the feature discarding mask (FDM) for a test face with random partial occlusions. Finally, the framework multiplied the FDM with original face features to eliminate corrupted feature elements from recognition.

Note that the above studies worked on either face mask detection or facial recognition with face covers, resulting in the models not applicable for a comprehensive face mask detection system that includes four features, face mask detection, mask type classification, mask position detection as well as facial recognition with face coverings. This work proposed four different models that solve the respecting features so that each model can be tuned separately to achieve best average accuracy and can be trained efficiently.

### 2.2. Technology Survey

Since the pandemic started, many companies have started their own development plan to adjust the detection process and identity recognition with the new normal. Following companies provide different commercial solutions on face mask detection and facial recognition with mask problems. Table 2 compares each product by its feature, application, business model, and cost.

LeewayHertz provides face mask detection applications for surveillance camera systems. The product provides a dashboard for real-time monitoring, interface to add face, identification information such as phone number, ID number. Moreover, it can raise the warning in real-time, so when it detects an individual who does not wear a mask, it will send a notification through the app. The product can detect the absence of a mask, identification behind the mask, but cannot identify the types or position of the mask. It does not require additional hardware to run the software and it can be integrated with any IP cameras or CCTV [10].

Tencent Youtu Face Recognition AI system was built in 2012. They concentrated on image processing, pattern recognition and deep learning areas leveraging the big data collected by the company’s social platforms. In order to solve the difficulties in accurately recognizing masked faces, Tencent updated their face detection and recognition algorithms. In terms of mask attribute recognition, the current Youtu algorithm can finely identify the following five situations: not wearing a mask, wearing a mask incorrectly and covering the mouth, wearing a mask incorrectly and covering the chin, wearing a mask incorrectly but not covering the face, and wearing a mask correctly. This attribute recognition is based on Youtu’s open-source FAN attribute recognition, and adds more attention mechanisms to the face positions where the mask may be distributed, which can accurately identify whether the face is worn correctly. At present, the accuracy of identifying whether a mask is worn or not exceeds 99%. Community managers, etc. can freely combine these categories according to the needs of different scenarios. At the same time, all enterprises and institutions can also use this technology to detect employees in a timely manner to ensure safe resumption of work [11].

CyberLink released a facial biometric solution for security, access control and health check called FaceMe. FaceMe is an integrated solution for real-time face mask detection, authentication, and temperature monitoring. It is also able to detect whether the mask is worn incorrectly and protects against spoof attacks. For individuals not wearing masks or with elevated body temperature, the system sends an early detection and warning notification through an app called U Alert [12].

TrulySecure from Sensory provides a real-time platform to detect identity using face, voice, or a fusion of face and voice. The application is also capable of protecting users from spoof attacks. Recently, Sensory announced that their TrulySecure product has been upgraded to include new ability, detect identity for people who are wearing face masks and accurately recognize cough and sneeze. TrulySecure allows app developers or OEMs to quickly add their face and voice biometrics to any mobile, desktop application, or devices [13].

## 3. Data Engineering

### 3.1. Data Collection

**Figure 1:**
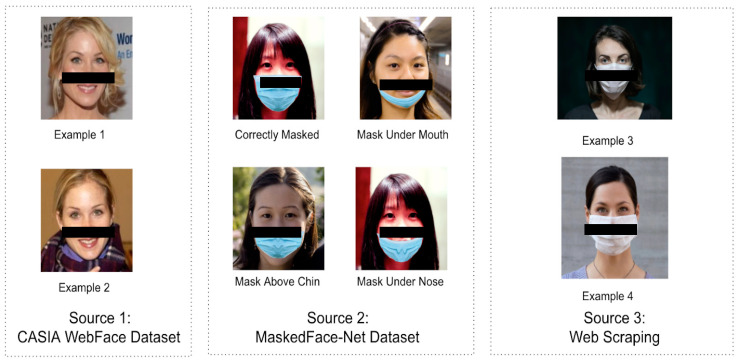
Sample Image Data from Multiple Sources.

The objective of the camera system is to accurately detect four different features: mask recognition, mask type, mask position, and authentication. To achieve this objective, data was collected from three different sources as illustrated in Figure 1: (1) CASIA WebFace Dataset [14] is the second largest open dataset for face detection problems. It contains 453,453 images over 10,575 identities. The dataset is categorized by unique identities in different folders. Within each folder, numbers of images for each identity are not the same. (2) MaskedFace Net is one of the largest datasets containing different types of masked face images. Different from other face recognition datasets, this dataset applied a mask face-to-face deformable model to simulate masks in different positions on human face images [15]. The net is divided into two categories: correctly masked face dataset with 67,193 images and incorrectly masked face dataset with 66,900 images. Within the incorrectly masked face dataset, it is further divided into three classes as uncovered chin, uncovered nose, uncovered nose and mouth. The amount of data in each group is balanced where 80% of the incorrectly masked face data with label uncovered nose and mouth, 10% with label uncovered nose and the rest 10% with label uncovered chin. (3) Web scraping technique is used for collecting masked faces in different types of masks, including N95, surgical, and homemade. The collected data are in various formats, sizes, and dimensions. Further steps will be taken to process web scraping data.

### 3.2. Data Preprocessing

**Figure 2:**
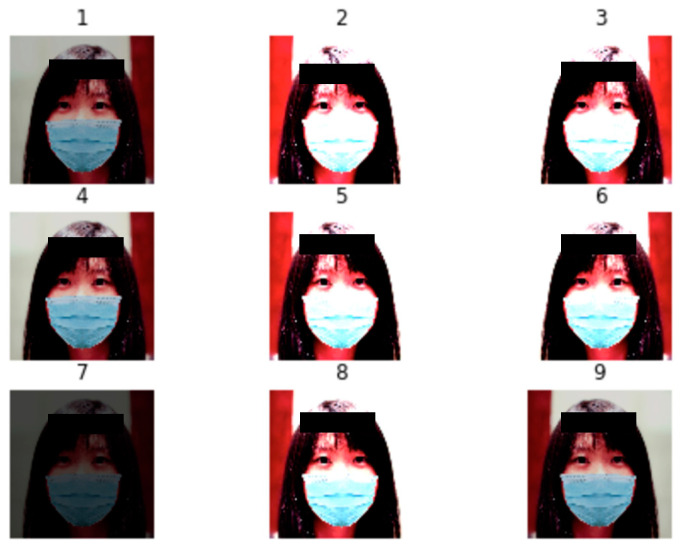
Sample Augmented Images.

In our project, the data pre-processing section is broken down into three steps: (1) data augmentation, (2) data simulation, (3) data preparation. The first step is data augmentation. For the raw data, four separate datasets regarding each feature are prepared. There are 1650 images for mask recognition, 2500 images for mask type classification, 9526 images for mask position classification, and 1000 images for facial recognition. As the quality and variety of training dataset is crucial to improve the performance and ability of the model to generalize, our goals for data augmentation are to expand the size and variety of the raw datasets, balance the dataset for each category, and reduce overfitting issues. To select the appropriate operations, the use cases of the system are taken into consideration. Since input data will be captured by camera in different angles and be taken under different lighting conditions, we simulated these two conditions with generation of mirror and different brightness images. Thus, we applied horizontal flip and brightness adjustment supported by Keras ImageDataGenerator where the brightness range is between 0.2 and 0.6. Figure 2 illustrates sample augmented images processed by ImageDataGenerator.

**Figure 3:**
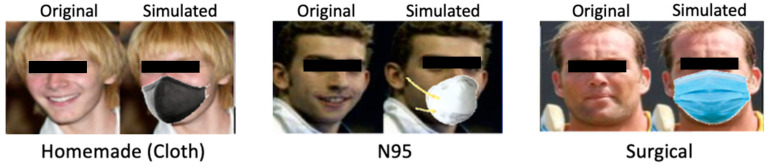
Sample Simulated Images.

The second step is data simulation. For the feature facial recognition with masks, 1000 images without masks were collected as raw data. In order to train the model for facial recognition with masks, the corresponding 1000 images with masks on need to be generated. MaskTheFace [16] is an open-source computer vision-based script masking tool that provides more than 90 different mask variation options. MaskTheFace can detect the faces in the picture, estimate the face tilt angle to locate the right face mask position, and add the user selected mask type on top of the input pictures. With the tool, we also expanded the size of the dataset for the mask type classification feature by adding different types of masks including surgical masks, N95, and cloth coverings on the raw data. Figure 3 illustrates sample images with the original one and the simulated one. 

The third step is data preparation. In this paper, we organized our mask recognition dataset, mask type dataset, and mask position dataset in terms of positive and negative. Negative subsets consist of the no mask images. For mask recognition dataset, positive subset is correct wear. For mask type dataset, positive subset includes three classes: N95, surgical, and homemade. For the mask position dataset, the positive subset includes four classes: mask above chin, mask under nose, mask under mouth, and correct wear. Table 3 describes the image numbers for each dataset in the three steps.

### 3.3. Training Data Preparation

To achieve a better performance for the camera system, different features are performed by different models and each model is trained separately. Respectively, we prepare the corresponding train, validation, and test datasets. For the mask recognition, mask types, and mask positions features, we randomly select 70% of the total images as train dataset, 15% of the total images as test dataset and the rest 15% of the total images as validation dataset. The train, test, and validation datasets are stored under three folders. In each folder, there are subfolders corresponding to different classes. Then we use Keras DataGenerator to read files directly from each directory in order to build the train, test validation data.

For the facial recognition dataset, each person will have his/her own folder, which contains at least 10 pictures of the same person wearing a mask. Images from all people in the dataset will be used in the training process, where in each person folder, 85% of the pictures are used to train the model, and the remaining 15% are used to test and evaluate the model performances.

## 4. Model Development

### 4.1. CNN

**Figure 4:**
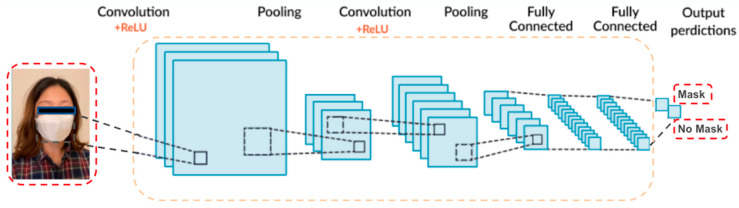
CNN Architecture for Mask Detection.

CNN is one of the widely used Deep Learning algorithms for image classification. Its ability to classify objects from 2-dimensional images is especially suitable for the mask detection from face images. Since CNN can learn the local patterns from the small window/location from an image, it will identify the location of the mask and eventually it will apply for the moving object as well during OpenCV. As seen in Figure 4, we build a CNN model with two convolutional layers, two max pooling layers, and two fully connected layers. The two convolution layers are set with 3 × 3 filters, no-zero padding, stride in (1,1) and ReLU activation function, the two fully connected layers are set with Relu and Softmax activation respectively. 

According to Younghak Shin et al. [17], CNN with three convolution layers outperformed HOG + CVG and SVM with respect to the evaluation metrics such as Sensitivity, Specificity, Accuracy, and Precision when the model is applied to identify the location or presence of polyps with colonoscopy datasets. The researchers also performed the same evaluation in grey and RGB, and the results were shown to be consistently higher for CNN.

### 4.2. Improved AlexNet

AlexNet is a type of Convolution Neural Network that is known for its image classification application, making it a good model for the detection of mask types. AlexNet is able to handle millions of images for training with low error rate according to the results at the ILSVRC-2012 image classification competition, making it ideal for the mask type features since they have large datasets. Compared to previous CNN models, AlexNet surpassed the capability of modern computer vision methods with further improvements made by its architecture. AlexNet consists of five convolutional layers and three fully connected layers. The output layers are two fully connected layers with 2048 units each, and are further connected to 1000 units of softmax layer which allows 1000 classes per requirement. The label-preserving transformations within the network also ensure classification accuracy. Running AlexNet requires multiple GPUs, which can slow down the training time; however, AlexNet’s architecture allows multi-GPU training by putting half of the model’s neurons on one GPU and the other half on another GPU [18]. This reduces training time while allowing a more complex model which contains 60 million parameters and 650,000 neurons to be trained [19]. This capability alone makes it ideal for mask type classification to ensure high accuracy with low error rate.

**Figure 5:**
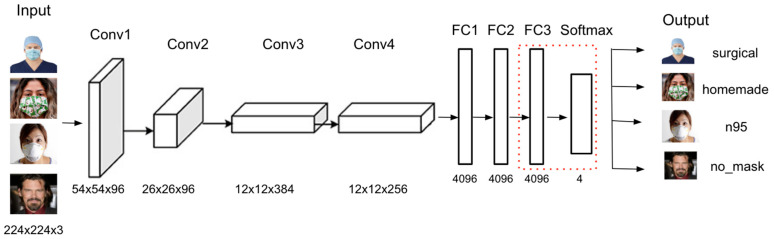
Improved AlexNet Model Architecture for Mask Type Detection.

However, the current AlexNet model has a slow computation time and can be easily overfitted. To enhance the performance and accuracy of the model, several modifications have been made and transfer learning was applied, which are shown through the AlexNet architecture in Figure 5. The transfer learning and modification are listed below:

Remove the fifth convolutional layer to improve computational time.

Add an additional fully connected layer with 4096 hidden units and apply RELU activation.

Add dropout layers in between the dense layers to prevent overfitting.

Modify the last output layer with softmax activation and set class labels to four classes instead of 1000.

### 4.3. Improved VGG16

VGG16 is a Convolution Neural Network designed for object detection and image classification. The model is proposed in the paper Very Deep Convolutional Networks for Large-Scale Image Recognition by researchers from University of Oxford. VGG16 was used to win the ILSVRC (ImageNet) competition in 2014 [20]. The ImageNet as a major computer vision benchmark dataset contains more than 14 million images belonging to 1000 classes. In the competition, VGG16 outperformed other models with 92.7% top-5 test accuracy [21].

VGG16 contains 16 layers with multiple repetitions of convolutional layers of 3 × 3 filter with pooling and stride 1, and uses the same padding and max pooling layer of 2 × 2 filter of stride 2. The input requires fixed size 224 × 224 RGB images, and the image passes to the first layer. With the arrangement of multiple stacks of convolutional layers, three fully connected (FC) layers followed by a softmax generates the output [22]. The first two FCs contain 4096 channels each, the third FC contains 1000 channels since it performs ILSVRC 1000 classifications. With the above architecture and training with ImageNet, VGG16 is capable of a maximum 1000 classes image classification. At the output layer, VGG16 uses a softmax layer having 1000 outputs per image category in the ImageNet dataset. The improved VGG16 replaced the output layer with one flatten layer, one dropout layer and two dense layers with sigmoid activation function having five outputs per image category. The weights are trained by the ImageNet dataset with more than 14 million images in 20,000 categories. The weights are then preserved in the pre-trained model and it gets 92.7% top-5 performance. Thus, VGG 16 is an advanced model to fulfill image classification tasks. The weights also help to avoid overfitting since sufficient training data is fed into the networks with 138 million parameters.

**Figure 6:**
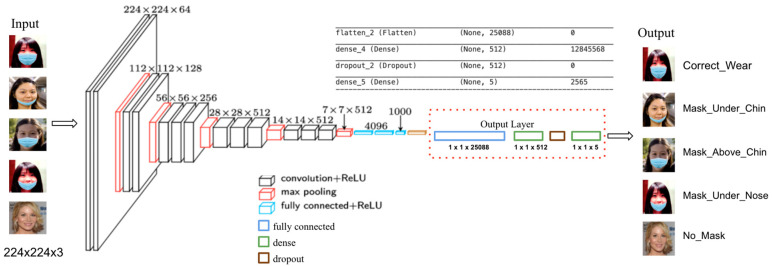
Improved VGG16 Model Architecture for Mask Position Detection.

The model weights of VGG16 are available on different platforms such as Keras. We will utilize VGG 16 model weights to perform feature extraction and classification. To better fit the model with our project data, we also performed transfer learning and fine-tuned the output layer. With the input images, the improved VGG16 model can classify the given input image into one of the predefined categories, which are mask under nose, mask under mouth, mask above chin, correct wear, and no mask. Figure 6 illustrates the modified VGG16 architecture. The transfer learning and modification are listed below:

Load the pre-trained VGG16 with freeze convolutional base and without output layer.

Add a fully connected layer with 512 hidden units and apply RELU activation.

Add a dropout layer with 0.5 dropout rate to avoid overfitting.

Modify the last output layer with sigmoid activation and set class labels to five classes instead of 1000.

### 4.4. Face Mask Facial Recognition Pipeline (FMFRP)

In order to recognize human identity with a mask on, we implement a facial recognition pipeline with a transfer learning algorithm. Our recognition system contains three main models: (1) a Multi-task Cascaded Convolutional Neural Network (MTCNN) as a face detector, (2) a deep network-based face recognition system called FaceNet to create face embedded vectors, and (3) a classifier (SVM/XgBoost) to predict the identity of a given picture. From the input image, MTCNN is used to create the bounding box pixels for the face position. FaceNet is a pre-trained deep convolutional neural network which creates face embedding then compares the faces in the embedding space to support decision making. FaceNet is well-known for using the Triple Loss function to encode images efficiently as feature vectors for rapid similarity calculation. However, using triplet loss makes FaceNet difficult to train due to it exotic architecture and complex calculation. These triplet losses sometimes would not contribute to the training and cause slower convergence rate. In a previous study, researchers have also explored the triplet semi-hard loss function, where they use all anchor positive pairs in a mini batch while still selecting the hard negatives and found out that this method was more stable and converged slightly faster at the beginning of the training [23]. In this study, our facial recognition pipeline is trained on masked face images, so we compared the pipeline performance on regular triplet loss from pre-train FaceNet model and triplet semi-hard loss function. Then we added different classifiers SVM and Xgboost after to extract face features and further compared the recognition accuracy.

**Figure 7:**
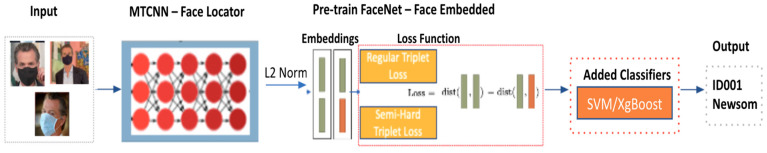
Face Mask Facial Recognition Pipeline.

Figure 7 shows the architecture of our facial recognition system. Image captures from input video will be passing through the system, and output will be the predicted user ID with name. In the training phase, images of the same person with mask on are fed into the network. The network maps these paired images to the embedding vectors and calculates center loss [24]. The L2 distance (Euclidean norm) is used in the loss function to imply similarity. These FaceNet embedding feature vectors will be fitted into the SVM and Xgboost classifier for identity classification. In the case of a new user that is not in our database, we take care of it by classifying anyone who has the highest-class probability less than 10% as unknown.

In the camera system, FaceNet is used to identify human identities with masks on their face. It is often used to extract high quality features from faces called embedded vectors, which are used to train the facial recognition system. The approaches include the following steps: (i) extracting faces from input image using Multi-Task CNN (MTCNN), (ii) extracting face features via face embedding tool FaceNet, (iii) fit the features into an SVM classifier to predict identity. Compared to other deep learning models for facial recognition, FaceNet uses an innovation called triplet loss function, which minimizes the distance between the same identity but maximizes the distance between different identities. This allows images to be encoded efficiently as feature vectors and fastens the similarity calculation and matching through distance calculations. Additionally, FaceNet is also able to achieve near or better than human level performance on standard face recognition dataset. Besides these strengths, FaceNet also has some limitations. If the input image contains a high level of noise such as blurry, missing pixels, brightness issues, the model performance will be impacted detrimentally. Another limitation of FaceNet is that the model works well with images of a single person, but it will have issues intermittently with images of multiple people.

### 4.5. Stacking Ensemble Model Learning Framework

**Figure 8:**
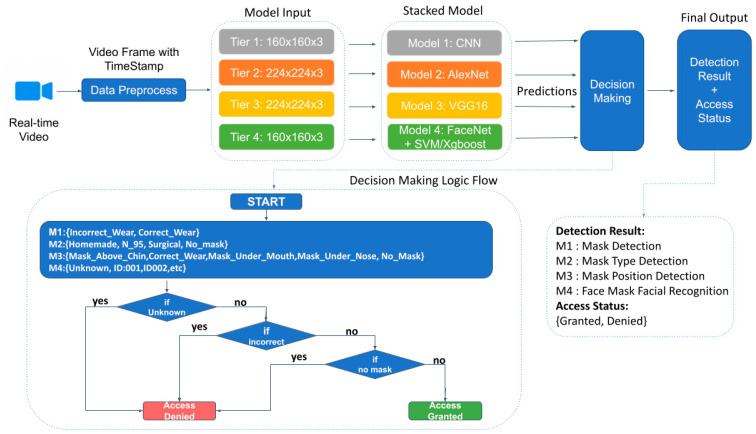
Stacked Model Working Flow.

Each model is trained and fine-tuned with respective datasets and achieved an average of 97% accuracy. Then, we designed a stacking ensemble model learning framework to acquire data, preprocess data, make decisions from a combination of predictions and generate final output with detection results and access status [25]. Figure 8 shows the stacking ensemble model architecture and the framework working flow. To acquire data, real-time video is processed by capturing video frames with timestamp and resizing to four tiers corresponding to required sizes for each model. Then input data feeds into the stacked model and the model generates a combination of predictions. The predictions include detection results for each feature, which are mask detection, mask type, mask position and identification. Besides the detection results, the predictions are used to determine access status as granted or denied by a decision-making process. If any of the prediction results contains any labels with no_mask, incorrect, or unknown, then the final output is presented with detection results and access status as denied. If the prediction contains labels as correct wear and identification, then the final output is presented with detection results and access status as access.

## 5. Model Evaluation with Case Study

Each part of the stacked models is trained and elevated independently with corresponding datasets as mentioned in the data engineering section. The TensorFlow deep learning framework is used to implement the training and evaluation phases. For the training phases, CNN, Improved AlexNet, Improved VGG16, and FaceNet are trained with corresponding data.

To evaluate the performance of the models, different combinations of performance metrics are selected based on the purpose of models. For standalone models, CNN, Improved AlexNet and Improved VGG16, accuracy curvers and loss curves under training, and validation data are plotted to evaluate the generalization ability of the model and to help fine-tuned the model in order to eliminate overfitting issues.

For integrated models, the face mask facial recognition pipeline with feature extractor and classifier is evaluated with experiment trails. The trails include the following specifications:

- Two loss functions (Regular Triplet, Triplet Semi-Hard)
- Two classifiers (SVM, Xgboost)
- Dataset splits to training and test with a ration of 85:15

### 5.1. Accuracy, Loss Curve and Confusion Matrix for CNN

**Figure 9:**
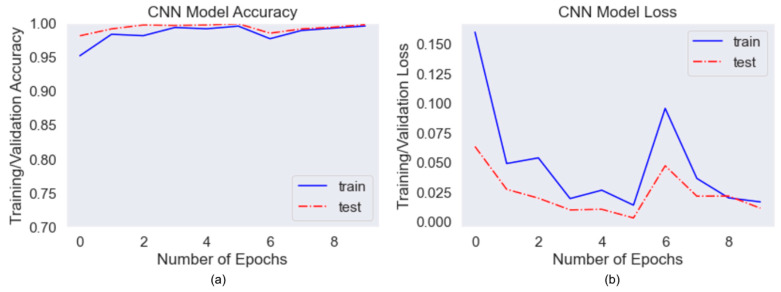
(**a**) CNN Accuracy Curve, (**b**) CNN Loss Curve.

CNN performed binary classification for mask detection feature. The input for training is image data with size 150 × 150 × 3 and the output is binary results with labels with_mask, without_mask. The model was trained with 8575 images and was validated by 1187 images. Figure 9 illustrates the accuracy and loss curves for 10 epochs. The train and validation accuracy curves converged and achieved an average of 97% accuracy. Even though both losses fluctuated, the gap between train and validation curves was acceptable. 

### 5.2. Accuracy, Loss Curve and Confusion Matrix for Improved AlexNet

**Figure 10:**
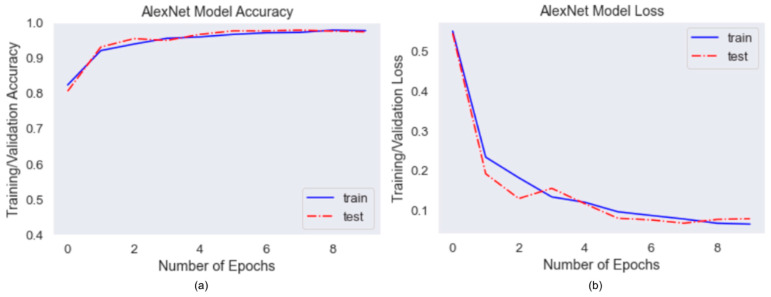
(**a**) AlexNet Accuracy Curve, (**b**) AlexNet Loss Curve.

Improved AlexNet performed mask type classification feature. The input for training is image data with size 224 × 224 × 3 and the output is multi-class results with four labels, surgical, homemade, N95, no mask. The model was trained with 7014 images and validated with 2104 images. In Figure 10, it showed that the model accuracy and loss curves converged pretty well after overfitting mitigation techniques. Initially, the graphs fluctuated consistently, but they became more stable after adding more images and doing more data augmentation. The average accuracy for validation data achieved 97%.

### 5.3. Accuracy, Loss Curve and Confusion Matrix for Improved VGG16

For the improved VGG16 model, we used image size 224 × 224 × 3 as input data. Since we built VGG16 by freezing the convolution base, we only trained the last output layer with our prepared data. 5 labels are assigned for all data, including Mask_Above_Chin, Mask_Correct, Mask_Under_Mouth, Mask_Under_Nose, No_Mask. Then we trained the last layer for 10 epochs with a batch size of 30.

**Figure 11:**
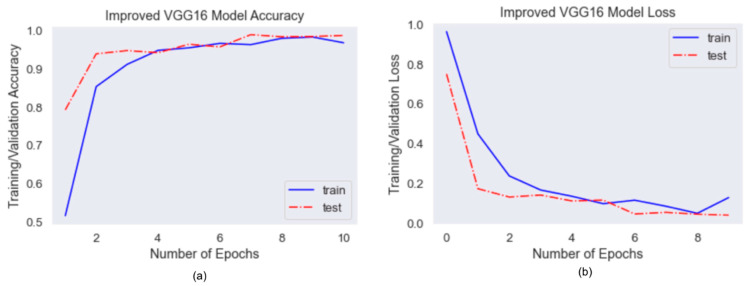
(**a**)VGG16 Accuracy Curve, (**b**) VGG16 Loss Curve.

Figure 11 shows the accuracy and loss curve on the train and validation dataset. The model performs well on classification of mask positions, both train and validation accuracy achieve 97%. From the loss curve we can tell that both train and validation loss are decreasing as epochs increase. It suggests that the chosen learning rate is performed well on the training data. The gap between train accuracy and validation accuracy suggests that overfitting issue is avoided with the improved VGG16 model. 

### 5.4. Accuracy and Performance Metrics for Face Mask Facial Recognition Pipeline

Our Face Mask Facial Recognition system contains the following tasks:

Locate the face position from the input images.

Extract facial embedded feature vectors using FaceNet.

Classify the input images to corresponding identities with classifier SVM and classifier XgBoost.

Then we evaluate our face mask facial recognition system on two main metrics: identification ability and verification ability. Table 4 shows the identification results by accuracy from using different loss functions and classifiers. Even though the triplet semi-hard loss function provides a better and more efficient loss computation when extracting face features, it does not work well in our study. Our face image inputs are covered with masks, which make the available face features less than normal. Respecting the classifiers, Xgboost shows higher accuracy which provides better confidence to classify the correct identity. However, it has issues with misclassification that brings down the accuracy. Therefore, we use regular triplet loss as the FaceNe loss function and choose SVM as the classifier for our Face Mask Facial Recognition system.

To evaluate the verification ability of the system, we chose a well-accepted statistical evaluation method for a biometrics-based authentication system, that is to confirm whether the presented biometrics are matching the enrolled biometrics of the same user. In our case, the biometrics we are evaluating is the behavioral characteristic, face features. Thus, the verification ability of our authentication system is determined by the face matching performance of the system. False acceptance rate (FAR) and false rejection rate (FRR) are two intrinsic parameters of a matcher. Threshold is another important parameter for evaluation. It determined the tradeoff between FAR and FRR of the system [26]. The threshold here for this system is the probability of the predicted class. To determine the best model threshold, we select the equal error rate that is the point where FAR = FRR.

**Figure 12:**
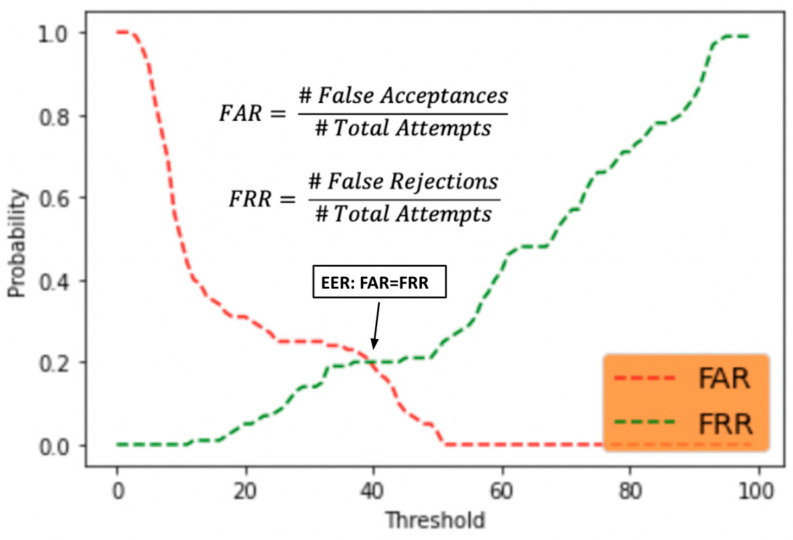
FAR and FRR curves for face mask facial recognition pipeline.

The curve of FAR shows the probability when the impostors can be identified as an original with given thresholds and the FRR curve shows the probability when the original gets rejected by different thresholds [27]. In our system, the optimal threshold is around 40% because it is the intersection point of FAR and FRR (Figure 12). Besides high accuracy of recognizing known users, our system has around 20% chance of falsely accepting the impostors or falsely rejecting the original one.

### 5.5. Case Study for Each Model

**Figure 13:**
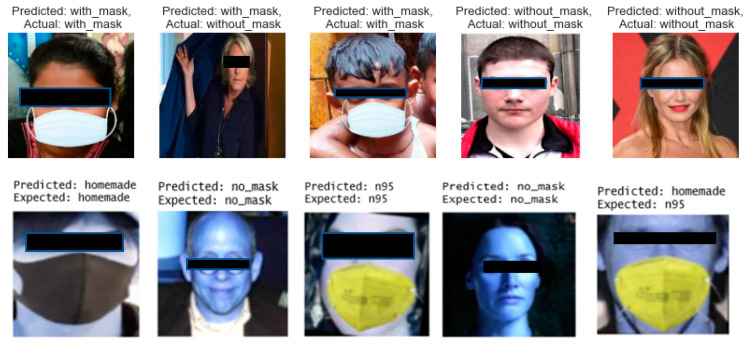

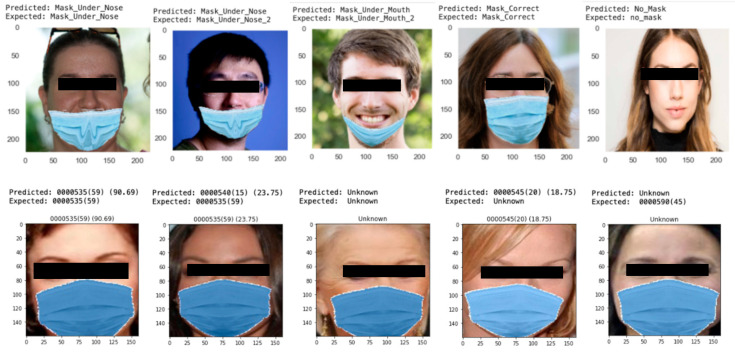
Testing Results.

In this section, we tested each model with unseen images in the training dataset to present the performance of each model. Figure 13 shows the testing results with predicted label and actual label each model in the order:

- CNN on mask detection
- Improved AlexNet on mask type
- Improved VGG16 on mask position
- Face mask facial recognition pipeline on user identification

## 6. The Overview of Spartan System

**Figure 14:**
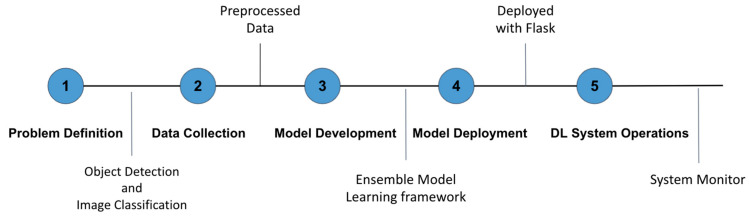
Life Cycle of Spartan System Design.

The Spartan Face Mase Detection and Facial Recognition system is an intelligent face mask classification and facial recognition system based on the developed intelligent models to support face mask detection, face mask type classification, face mask position classification and facial recognition. The system uses a comprehensive and diverse human face dataset, which includes people’s images with different types of masks, with different positions of mask and with identification. As illustrated in Figure 14, the full-life cycle of building the system includes problem definition, data collection, model development, model deployment, and DL system operations.

First, we framed the face detection and identification problems to object detection and image classification. Then we collected 13,550 raw images and augmented them to 381,550 images. Regarding model development, we leveraged transfer learning techniques on multiple deep learning models for each classification problem. To integrate the developed DL models, we implemented a stacking ensemble model learning framework for combined prediction results. By splitting the face mask detection job in different tasks and executing them simultaneously in parallel, significant boosts in integrated model performance are achieved. To deploy the models, we used the OpenCV videocapture function to divide videos into frames and used Haar-cascade classifiers to detect faces from the computer’s built-in cameras [28]. Since each feature requires different input sizes, we resized the captured images. The resized images will be fed into each feature in parallel, and generate multiple combinations of predictions. Then, we used the Flask framework to support the ensemble deep learning models. For DL system operations, we build the infrastructure with the training platform and a server with core DL algorithms. The server can accept requests from the user side and respond with predictions on the interface for administration.

The Spartan Face Mase Detection and Facial Recognition system is designed to detect masks, classify mask type and position and recognize personal identity with masks on. The system will be operated by admin users where they can monitor real-time results on the interface. The system targets are users who will be captured by cameras and their images will be analyzed by the server. Considering the business and resource availability resources, the Spartan system serves with online prediction and edge computing [29]. The tasks of face detection and facial recognition require immediate response when requests come. It also requires that when image samples are generated from video acquisition and video processing, predictions are needed to align with the image samples. The advantages of online prediction are real-time, low latency and no limitations on prediction counts. Besides, the Spartan system is powered by edge computing where all predictions and computations are finished on edge devices such as phones, browsers, laptops and tablets. Since the Spartan system is lightweight and does not require cloud storage or cloud computing, edge devices with limited memory, compute power and energy are eligible for the Spartan system operation in which the system performance will not be compromised due to the limitations. With edge computing, the system can work stable without internet connections, thus internet latency will not affect system performance. In addition, there are fewer concerns about system security and user privacy when the system is running offline. Overall, the Spartan system provides comprehensive face detection features with real-time predictions, low cost, low latency, and less concerns on privacy issues.

### 6.1. System Operations

**Figure 15:**
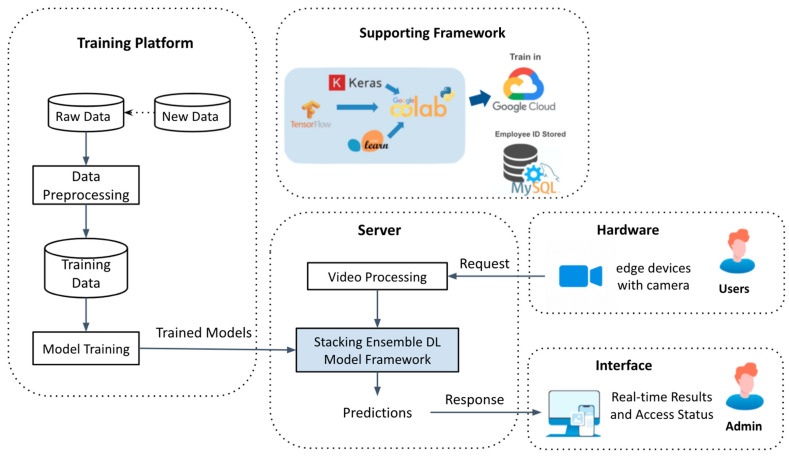
Block Diagram of Spartan System.

The Spartan system contains five main modules which are training platform, server, hardware, interface, and supporting framework. The block diagram of the Spartan System is shown in Figure 15.

#### 6.1.1. A. Training Platform

The key algorithms of the Spartan system are established on the training platform, where it integrates the complete deep learning models training pipeline. The DL models can continually improve based on new data by offline learning. The complete pipeline includes data pipeline [30] and model training and model specification. Since raw data are categorized to corresponding models and stored separately, personal identifiable information can be handled properly with admin access. Models are trained separately on Google Colab so that hyperparameters of each model can be tuned for different use cases and achieve optimal performance. Model specifications are unit tested with random inputs to detect any errors in the code. They are also reviewed so that proper versions of model training code can be retrieved for faster re-training.

#### 6.1.2. B. Supporting Framework

The supporting framework for the system involves integrated service tools based on open-source tools. Data pipeline and model training are performed with Keras, Tensorflow, and scikit learn deep learning packages. The integration of four models as well as training data image processing are organized in Google Colab. With respect to the training data storage, all data is saved both locally and in Google Cloud. With the integration of Colab and Google cloud, large datasets can be retrieved and trained for complex improved deep learning models.

**Figure 16:**
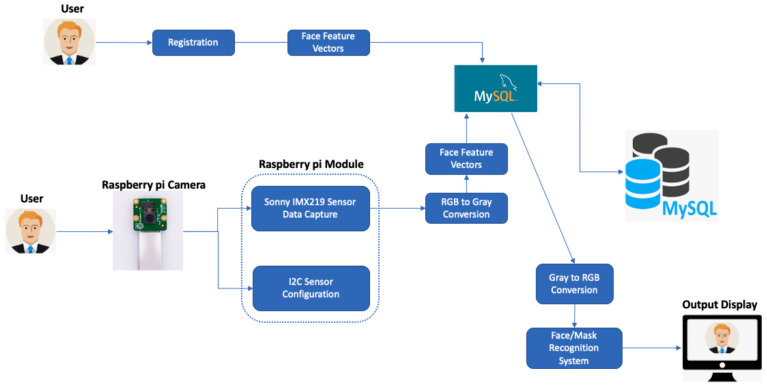
Employee Data Operation Flow.

For the purpose of facial recognition, large and confidential employee data with personal identifiable information for the organizations are stored and managed by MySQL database. We designed a data operation flow for those employee data using Raspberry Pi camera module and Sony IMX219 Sensor. Figure 16 illustrates the data working flow. First, users need to register their identity. The system will extract the face features and send it to MySQL server to store it. When the user stands in front of the camera, the Sony IMX219 Sensor will capture multiple user’s images [30]. These images will be converted to Grayscale before sending to the MySQL server to store in the database. Processing three-channel color images takes three times as long as processing single grayscale images. Therefore, grayscale conversion will help us cut the processing time, which is the most important thing in a facial recognition system. In the MySQL database, images are saved in the table as a Binary Large Object object (BLOB). Table contains user ID, user first name, user last name, user face images as BLOB objects. Data in the table is maintained and updated in real-time. When a new input comes in, our system will scan through the entire table for an identity match. If there is a match, new pictures will be added to the table for the existing user by ID.

#### 6.1.3. C. Server

Server of the Spartan system is powered by stacking ensemble DL model framework and is connected with hardware and interface. When the server receives a request from edge devices, the request is first processed by the video processing part. The streaming videos are captured to frames, and OpenCV Haar-cascade classifiers detect human face features from the frame images. Then images are normalized to required size and pixels before feeding into the ensemble model framework. With the video processing part, real-time videos are transferred to images with timestamp and resized images to different tiers. The stacking ensemble model learning framework integrates four deep learning models. To deploy the system on a local server or cloud server, Flask is implemented with the video processing and model framework. The framework generates predictions for each frame of the streaming video. The requests from hardware are responded with real-time predictions, timestamps, and videos. The infrastructure of the server ensures the DL pipeline is integration tested for correct function and model quality is validated on an offline environment to inspect any errors before serving the real request.

#### 6.1.4. D. Hardware

Hardware for the system are edge devices cameras, such as built-in computer cameras, external cameras or cameras with Raspberry pi [31]. At the gate of controlled and restricted buildings, the Spartan system runs on the edge devices. Visitors with masks should face the camera in order to allow the system to detect the faces and generate predictions.

#### 6.1.5. E. Interface

On the admin end, the detection results are displayed with a user interface (UI) showing real-time results with timestamp and access status. The interface is built with python, CSS, and HTML. Details of the interface are discussed in Section 6.3.

### 6.2. High-Level Data Analytics

High-level data analytics process is designed to maintain the system operating and provide reliable predictive results. The data analytics for the system is prepared in four aspects: Elicitation, analysis, specification, and validation and verification [32].

In the elicitation process, only mask-related face characteristics will be processed and trained by the ML functions. On the other hand, the input images captured by webcam will only be used to generate mask-related classification results and identification results.

For the analysis stage, we designed several performance measures to evaluate the system. When we evaluate the models, we mainly use accuracy, misclassification rates and predicted probabilities of particle labels. To evaluate the performance of the system, we record the error rate and processing time. Error is recorded if the display results are not consistent with the actual situations in mask type and position classification and facial recognition. Runtime is recorded from the start of the detection process to the result display stage during the operations of the system.

The requirements specification includes the data requirements and the use of data. Training data are collected and processed under the same format, size, and resolutions. The input data for the system is captured by a webcam and processed by the video acquisition module. Distance to the camera and lighting conditions are required in order to capture clear images to process. With respect to the use of the data, regulations and constraints regarding the use of data varies under different circumstances. Hospitals, schools, and companies should follow the community rules when storing, analyzing, and distributing the data.

To ensure that training data corresponds to the real data, requirement validation needs to be performed continuously. Monitor and analysis of runtime data are essential for maintaining the performance of the system. Thus, the ML systems need to be retrained regularly to adjust to the recent data.

### 6.3. User Interface and User Cases

**Figure 17:**
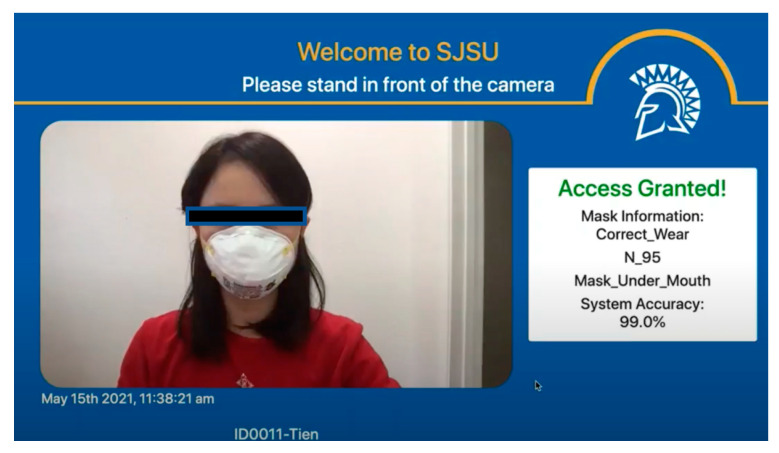

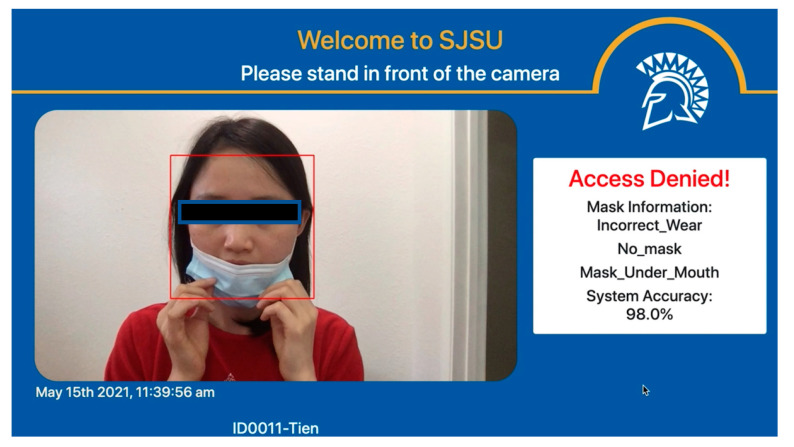
Screenshot of System User Interface.

Figure 17 illustrates system user interface (UI) design with two sample outcomes. The design of UI consists of the following elements:

- SJSU title, logo and system name
- Real-time video with timestamp
- Real-time prediction results display with access granted or access denied

**Figure 18:**
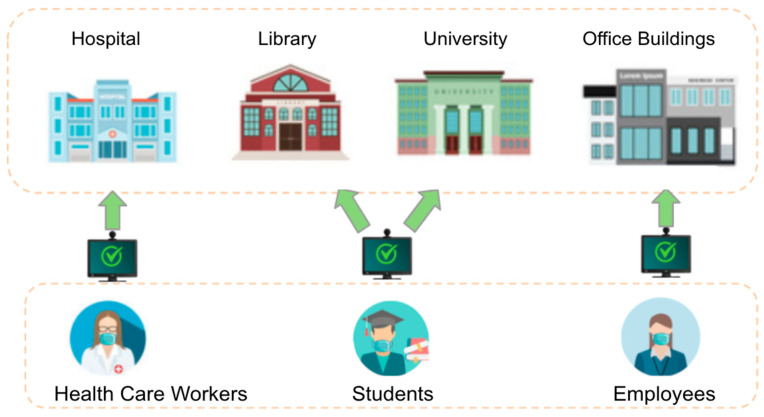
Use Cases of Spartan System.

The application of the Spartan system can be in many different industries. Figure 18 illustrates four possible use cases including hospital, university and library, office buildings. Under controlled environments which require gated access such as companies, university facilities, and hospitals would be required to check not only the presence of masks but also matching identity of users with the existence of the identity in the database. The importance of the feature that checking mask type can be beneficial for hospitals setting because the employees of hospitals might be required to wear only certain types of masks such as N95, KF94 or surgical masks etc. In addition, checking identity behind the mask is a critical feature because users do not need to take off a mask to verify their identity.

For a less strictly controlled environment such as a grocery market, shopping centers or airport, they can use the outcome of this project to check only the presence of masks regardless of mask types. However, for these types of environments, the feature of checking the position of the mask could be critical because all customers have to wear a mask correctly. Since the pandemic started, almost all retail stores put one designated person to check if customers wear a mask or not. What the outcome of this project can do is substitute the designated employee to check the presence of masks for all customers and also can raise an alarm if there is any incorrected wear mask or absence of mask to the store.

## 7. System Quality Evaluation and Performance

Correctness of the model is evaluated by probability for each classification. The probability is different for each model and each class. Run-time performance is evaluated by the runtime seconds for each model. We used getTickCount and getTickFrequency from OpenCV to record the runtime for each iteration. Runtime performance varies by different device processors and device cameras. Table 5 compares the feature average runtime and device specs.

Mask Detection: the average run time is 0.0031 s for 10 iterations.
Mask Type Detection: the average run time is 0.005947 s for 10 iterations.
Mask Position Detection: the average runtime is 1.501 s for 10 iterations.
Face Recognition with Face Mask: the average runtime is 0.054 s for 10 iterations.

Besides the runtime evaluation, system detection performance is also evaluated by real-time video with accuracies under different testing cases. Each feature of the system is tested with real-time videos.

**Figure 19:**
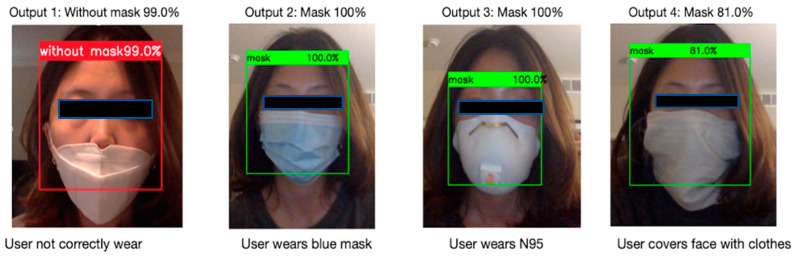
Face Mask Detection in different scenarios.

Figure 19 shows the system detention accuracy for face mask detection. There are four testing cases: mask not correctly worn, user wears blue mask, user wears N95, user covers face with clothes. Green boundary represents label with mask, and red boundary represents label without mask.

**Figure 20:**
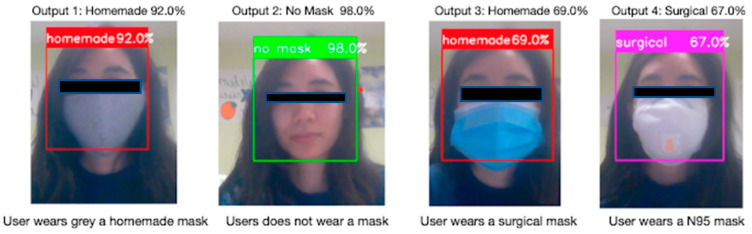
Mask Type Detection in different scenarios.

Figure 20 illustrates testing results on the feature mask type detection. There are four testing cases: user wears a grey homemade mask, user does not wear a mask, user wears a surgical mask, user wears a N95 mask. Each label is represented with a different color and corresponding detection accuracy.

**Figure 21:**
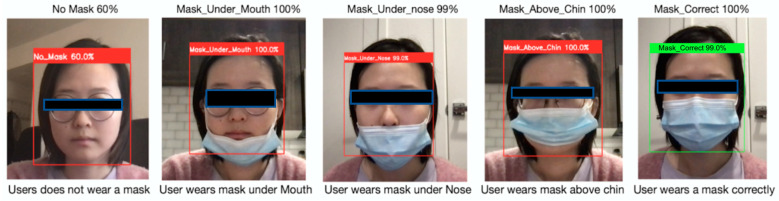
Mask Position Detection in different scenarios.

Figure 21 illustrates testing results on the feature mask position detection. There are five testing cases: user does not wear a mask, user wears a mask under mouth, user wears a mask under nose, user wears a mask above chin, user wears a mask correctly. Label Correct wear is represented with green boundary, and the rest labels with other positions are represented with red boundary.

**Figure 22:**
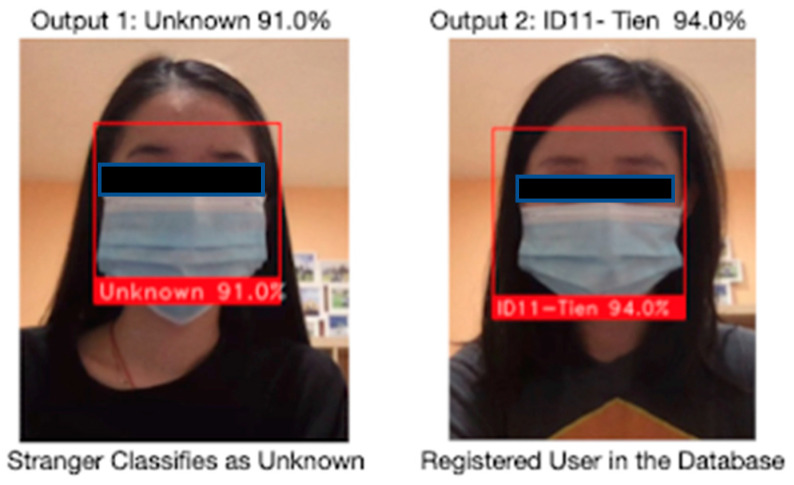
Face Mask Facial Recognition in different scenarios.

Figure 22 illustrates testing results on the feature face mask facial recognition. There are two testing cases: unregistered user and registered user. Unregistered users are defined as user facial information is not stored in the database and registered users are users with their facial information stored in the designated database. The system will label unregistered users as unknown and recognize registered users with stored user name and user id.

## 8. Conclusions and Future Work

Under the spread of COVID-19 pandemic, wearing a protective face mask has become a normal and requirement for many public services, colleges and essential business providers. Both WHO and CDC also stress on the importance and effectiveness of wearing correct masks for personal and public health. Therefore, face mask detection and facial recognition with a mask are essential for our society. To serve the above purpose, this paper proposed a Spartan Face Mask Detection and Facial Recognition with Mask System for access and health check under the pandemic. This comprehensive system provides mask detection, mask type detection, mask position detection, and face mask facial recognition. A stacking ensemble model learning framework is presented as the core algorithm. There are four parts for the stacking model: CNN, improved AlexNet, improved VGG16, and facial recognition pipeline. Different training and validation approaches are applied for each part of the model and four datasets are used. Based on the testing results, each model performed relatively well and were able to accurately detect their classes for each feature. An average of 97% accuracy is achieved with proposed models and it indicates that the models can be integrated together and tested on real-time videos. The results show the balance of limited computing resources and high performance.

Along with the Spartan system, an end-to-end solution is provided with video acquisition, database design and high-level data analytics. The system and the solution can be easily used by small businesses, organizations and universities with minimum cost under the COVID-19 and help to practice social distancing. One of the possible future works is extending the system with more features such as temperature check and social distance check so that the system can be used in more scenarios.

## Figures and Tables

**Table 1:** Literature Survey.

ID	Focused Problems	Algorithms	Accuracy	Data (Images)	Labeling
[3]	Face Mask Detection	Resnet 50 DT and SVM	99.5%	RMFD (95 K) SMFD (1570)	Manual
[4]	Identity Recognition w/Mask	Fisherfaces and PCA	88%	Bosphorus 3D Face Dataset (4 K) FRGC Dataset (50 K)	Manual
[5]	Identity Recognition w/Mask	Gabor wavelets and PCA	85%	Olivetti Research lab (400)	Pre-labeled
[6]	Face Mask Type Detection	ResNet ssd_mobilenet_v2_coco	97%	Google Images (800)	LabelImg
[7]	Face Mask Type Detection	LLE-CNNs	76.4%	Google (300 K)	Manual
[8]	Proper Mask Wear Detection	Facial Abstraction Net	91.8%	The Helen Dataset (2330)	Facial Attribute Prediction Net
[9]	Proper Mask Wear Detection	Siamese Networks Trunk CNN	98.2%	CASIA-WebFace dataset (494 K)	Divide Pictures into pixels

**Table 2:** Technology Survey.

Product	Description	Face Mask Detection	FaceMe	Youtu AI System	TrulySecure
Company	AI Functions	LeewayHertz	CyberLink	Tencent	Sensory
Features	Protects against spoof attacks	No	Yes	No	Yes
	Real-Time Detection	Yes	Yes	Yes	No
	Early Detection & Warning	Yes	Yes	Yes	No
	Authentication via face & voice	No	No	Yes	Yes
Applications	Face Recognition	Yes	Yes	Yes	Yes
	Mask Detection	Yes	Yes	Yes	Yes
	Mask Position	No	Yes	Yes	No
	Authentication	No	Yes	Yes	Yes
Business Model	Provide API	Yes	No	Yes	No
	Provide SDK	No	Yes	Yes	Yes
	Provide Camera	No	No	No	No
Cost	API	Tier Pricing	N/A	Tier Pricing	N/A
	SDK	N/A	Tier Pricing	Tier Pricing	Voice detection (TSSV 2.0 SDK, $2500 *)

* Additional licenses required for additional features and size of systems.

**Table 3:** Image Numbers for Each Dataset.

Dataset	Raw	After Augmentation	After Simulation
Mask Detection	1650	11,425	11,425
Mask Type	2500	11,123	12,123
Mask Position	9526	9526	9526
Facial Recognition	1000	1000	2000
Total	14,676	33,074	35,074

**Table 4:** Model Accuracy by Classifiers.

Classifier	Train Accuracy	Test Accuracy
SVM	100%	97%
XgBoost	100%	88%

**Table 5:** Run-Time Performance Comparison.

Feature	Device Specs	Average Run Time (s)
Face Mask Detection	Macbook Pro Processor: 2.9 GHz Dual-Core Intel Core i5 Memory: 16 GB 1867 MHz	0.003
Mask Type Classification	Dell XPS 13 Processor: 10th Generation Intel^®^ Core™ i7-1065 G7 Memory:16 GB 4267 MHz	0.005
Mask Position Classification	Macbook Pro Processor: 1.4 GHz Quad-Core Intel Core i5 Memory: 8 GB 2133 MHz	1.501
Face Mask Facial Recognition	Macbook Pro Processor: 2.9 GHz Quad-Core Intel Core i7 Memory: 16 GB 2133 MHz	0.054
Integrated System	Macbook Pro Processor: 2.9 GHz Quad-Core Intel Core i7 Memory: 16 GB 2133 MHz	0.614

## Data Availability

Data available Data available in a publicly accessible repository that does not issue DOIs. Publicly available datasets were analyzed in this study. This data can be found here: https://github.com/cabani/MaskedFace-Net (accessed on 1 July 2021).
